# Social comparison impacts stimulus evaluation in a competitive social learning task

**DOI:** 10.1371/journal.pone.0234397

**Published:** 2020-06-25

**Authors:** Rebecca Burnside, Markus Ullsperger

**Affiliations:** 1 Department of Neuropsychology, Institute of Psychology, Otto-von-Guericke University, Magdeburg, Germany; 2 Center for Behavioral Brain Sciences (CBBS), Otto-von-Guericke University, Magdeburg, Germany; Temple University, UNITED STATES

## Abstract

When we perform an action, the outcome that follows it can change the value we place on that behaviour, making it more or less likely to be repeated in the future. However, the values that we learn are not objective: we interpret the outcomes that we receive for ourselves relative to those that share our environment, i.e. we engage in social comparison. The temporal dynamics of physiological responses to stimulus valuation in social learning tasks are poorly understood, particularly in human participants. Therefore, we recorded stimulus-locked event-related potentials with 64-channel EEG to examine stimulus valuation, following the design of a study previously used in macaques. Pairs of participants performed a social learning task in which they received outcomes sequentially for a presented stimulus (partner first) by pressing a button in response to a cue. There were two conditions: one in which stimulus values varied for the participant but output a constant rate of reward for the partner (self-variable blocks), and another condition in which this payout was reversed (other-variable blocks). We then measured participants’ self-reported competitiveness. Approximately 200 ms post-stimulus, an ERP related to stimulus evaluation and attentional processing appeared to encode own stimulus value in self-variable blocks. In other-variable blocks the same pattern of activity was reversed, even though the value of the stimulus for the participant did not depend on the stimulus presented. Outcome-locked analyses further showed that attention dedicated to the partner’s outcome was greater in more competitive participants. We conclude that subjective stimulus value can be reflected in early stimulus-locked ERP responses and that competitive participants may be more invested in their own performance relative to the other player, hence their increased interest in the outcome of their partner.

## 1. Introduction

In environments in which resources are scarce, humans and other animals compete for the same goods. In such contexts, the success of a competitor in locating food or water can be interpreted as a reduction in one’s own ability to obtain the same resource. In other words, our own receipt of reward and the observation of reward received by others is subject to social comparison [1]. In reinforcement learning (RL) paradigms, in which participants learn stimulus-outcome contingencies to maximise their reward, there is evidence that social context impacts the interpretation of outcomes. The feedback related negativity (FRN), for example, is an event-related potential that is sensitive to social context effects. The FRN appears 250–300 ms after outcome presentation and is thought to encode a prediction error (PE), i.e. a discrepancy between an expected and actual outcome received by a participant on a given trial. The signal appears to originate in the posterior medial frontal cortex (pMFC) and is typically greater for negative compared to positive outcomes [2], which can be enhanced further if a loss is received in the presence of the gain of another participant [3]. The encoding of valence can even reverse direction entirely (positive outcomes > negative outcomes) in response to observed outcomes if the target of observation is a competitor [4, 5]. In another study, the ‘other-FRN’ (i.e. the FRN in response to the outcome of the other player) was greater for observed than own outcomes in a gambling task, but only for participants in a competitive social condition [6], with the explanation that greater attention to the performance of a competitor aids one’s own performance. At present the direction and extent of other-FRN effects in social contexts are not always consistent between studies, and while the other-FRN is thought to reflect a prediction error, there is evidence that the other-FRN may not necessarily reflect the same computation [7, 8].

Recent research in non-human primates has shown that social comparison not only impacts outcome processing, but can also alter reward expectation at the point at which a stimulus is presented [9]. In a Pavlovian social learning task played by two monkeys, the authors found that an increase in reward probability for a partner monkey led to a decrease in reward expectation behaviour (licking for juice) in the player monkey, even though the reward the player received was at a constant rate. In the brain, ‘partner type’ neurons in the pMFC decreased their rate of fire in response to an increase in reward probability for the partner monkey, again despite a constant rate of reward for the player monkey. Stimulus processing is an integral component of learning from reinforcement, but value related neural processing at the time of stimulus presentation has not been studied extensively in social tasks with human participants [10].

What we do know is that to learn the value of an item or object, it should prompt some representation of the expected outcome to be obtained from it, i.e. its incentive value [10, 11]; and that this expectation of reward can be reflected in behavioural and physiological measures, such as reaction time (RT), skin conductance and neural activity. RTs to reward-predicting stimuli tend to decrease as a function of their reward probability and magnitude [12] and individual differences in reward sensitivity have been reflected in the activity of the motor cortex and correlate with RT [12]. Therefore, the motor cortex may integrate subjective stimulus value to motivate task performance. Skin conductance responses (SCR), meanwhile, increase for stimuli associated with punishment relative to neutral stimuli [13]. This makes RT and SCR useful measures to gain insight into subjective stimulus values in human participants.

Another measure that can reflect response-predictive and incentive value is the P200 component. The P200 is an event-related potential (ERP) maximal at fronto-central electrode sites between 150 and 250 ms after stimulus presentation [14]. There is evidence that the P200 reflects early dedication of attentional resources to stimulus evaluation and/or indexes task relevance of visual stimuli [15, 10, 16, 17, 11]. One study that has examined the timecourse of stimulus evaluation in human participants found that response-predicting stimuli provoked greater P200 responses compared to non-predictive stimuli [10]. Another study found that, after a choice is made but prior to feedback presentation, the P200 component reflected differences in reward expectation [18].

**Fig 1:**
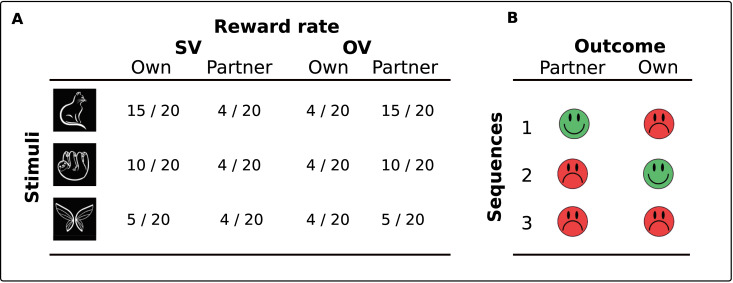
Reward output rates and reward sequences. (A) The table depicts the rate of reward (gains) that would be associated with each stimulus for the player and their partner in an SV or OV block. (B) The table depicts the three possible sequences in which rewards are presented. To make the task competitive, the player and their partner are never rewarded on the same trial.

In the present study, we examine the timecourse of stimulus and outcome processing in a social learning task (see [9]). Participants were presented with one of three stimuli on each trial in two block types: one in which stimulus reward contingencies varied for the participant but remained constant for another player (self-variable [SV]), and one in which this reward scheme was reversed for the player and their counterpart (other-variable [OV]). Participants were cued to reveal the feedback of the other player, then after a second cue their own feedback, by responding with a button press. Their aim was to learn the value of the stimuli for themselves and the other player over the course of each block to earn money. Importantly, a positive outcome for the other player and participant never appeared consecutively. This meant that although participants received a constant rate of reward for each stimulus in OV blocks, after seeing a loss for the other player on a specific trial, the participant could expect a gain for themselves on the same trial and vice versa (Fig 1). We recorded RTs, stimulus- and outcome-locked ERPs, and SCR from participants as they completed this task. To gain insight into participants’ explicit stimulus value estimates, we also asked participants to make stimulus value estimates for each image for themselves and the other player every 15 trials. At the end of the experiment, participants completed a competitiveness questionnaire.

Our hypotheses were divided into two categories: outcome related and stimulus value related. Here we outline the outcome related hypotheses. First, we expected a typical own FRN (losses > gains) in response to participants’ own outcomes in the SV condition in line with existing studies [2]. Second, we hypothesised that if competition increases attention to outcomes received by another player, similar to the study by Yu et al. [6], the other-FRN could increase in amplitude in line with participants’ trait competitiveness when observing the partner receive an outcome in OV blocks. We did not have a more general hypothesis related to self- versus other-FRN amplitude, because rewards were allocated sequentially and the other player always received their outcome first.

Stimulus-value related hypotheses were as follows: in terms of behavioural performance, if RT varies according to incentive value, (e.g. [12]; [19]), we would expect participants to be slower to reveal the other player’s feedback on trials in which there is a higher chance of reward for the other player. This would be consistent with a ‘your loss is my gain’ social comparison type valuation process, in which participants place higher subjective value on stimuli that are perceived as ‘bad’ for the other player. We expected this effect to be enhanced in more competitive participants on the basis that they would be more invested in their competitor’s relative rate of reward.

We expected to see a similar pattern in the physiological measures of incentive value to the RT data. We hypothesised that, if the P200 reflects task relevance and/or incentive value of a presented stimulus, as demonstrated in a range of studies [10, 16, 17, 11], then we would expect larger amplitudes in response to stimuli with a higher rate of reward for the participant in the SV condition. We would also expect that this pattern would reverse in the OV condition, i.e. that the stimulus with the highest reward rate for the other player would provoke a larger P200 amplitude than the lower reward rate stimulus. Similar to Yu et al. [6], we expected competitive participants to be particularly attentive toward the outcomes received by the other player. In this case we could expect a greater other-FRN when observing the partner receive an outcome in OV blocks, which increases in line with participants’ trait competitiveness. Finally, the SCR results were expected to correspond with the P200 results, such that greater SCR amplitude would be associated with low reward rate stimuli in SV blocks, a reversal of this pattern in OV blocks, and an increase in line with participants’ trait competitiveness.

## 2. Method

### Participants

Thirty-one healthy participants (female = 18, right-handed = 28, M_age_ = 25.90) were recruited from the Otto-von-Guericke University (OvGU), Magdeburg. All subjects provided written informed consent to participate in the task and were remunerated for their time, with money or course credits. Ethical review and approval for this research was provided by the ethical review committee of the Otto-von-Guericke University (no. 23/14).

### Experiment setup

Participants were recruited in pairs to complete a probabilistic social learning task adapted for humans from Noritake et al. [9]. After providing their consent to participate, they were taken to two separate acoustically and electrically shielded cabins within the same laboratory and prepared for EEG and skin conductance recordings. They were informed that we were investigating neural activity related to active and observational learning and that one participant would play as the ‘actor’ and perform all actions in the learning phase of the task, while the other player observed. Both players would then have their stimulus value knowledge assessed in test trials at a later point and would be allocated bonus points for correct answers. In fact, participants performed the task separately and were both designated the ‘actor.’

### Learning trials

The task consisted of eight blocks of 60 trials (total = 480 trials), in which three stimuli were presented individually 20 times each. Stimuli were white line drawings of animals or objects presented on a black background in the centre of the screen. At the end of each block, the stimuli were replaced by a new set of three images. On every trial, participants received two outcomes for the presented stimulus, one for the other player and then one for themselves. The probability that participants would receive good feedback depended on the type of block that was being completed. In self-variable (SV) blocks, a stimulus could be good (75%), neutral (50%), or bad (25%) for the participant, but had a constant reward output (20%) for the other player. In other-variable (OV) blocks, the reward structure was reversed, such that the participant received a constant reward output (20%) and the reward output for the other player was stimulus-dependent (good = 75%, neutral = 50%, bad = 25%). There were four SV and four OV blocks. Block order, reward output, and the order in which stimuli appeared within a block were pseudorandomised. Pseudorandomisation ensured that no stimulus could be rewarded more than 5 times consecutively and that no stimulus would appear more than three times consecutively.

In accordance with Noritake et al. [9], there was a competitive element to the allocation of the outcomes. The purpose of the competitive aspect was to mimic competition for resources that arises ecologically. There is evidence that resource scarce environments like these provoke competitive behaviour in animals [20, 9] but also human participants [21, 22]. On any given trial, outcome allocation was restricted to the following possible sequences: gain-loss, loss-gain, and loss-loss. In other words, it was not possible for both the active and observing player to receive a good outcome for the same stimulus on a single trial. A consequence of the design is that on a trial in which the other player is rewarded, the expectation of reward for the participant on that specific trial should become zero. If, on the other hand, the other player is unrewarded on a given trial, the expectation of reward for that trial for the participant increases (see Fig 1 in the supplementary materials of [9]). Nonetheless, in OV blocks there would be no difference in the total number of rewards allocated to the participant across the block per stimulus (4/20 rewarded). Therefore, at the time that the stimulus is presented in these block types, the actual probability of reward is the same regardless of the stimulus presented. We were particularly interested in the ERPs and SCRs locked to the presentation of this stimulus.

### Learning trial sequence

**Fig 2:**
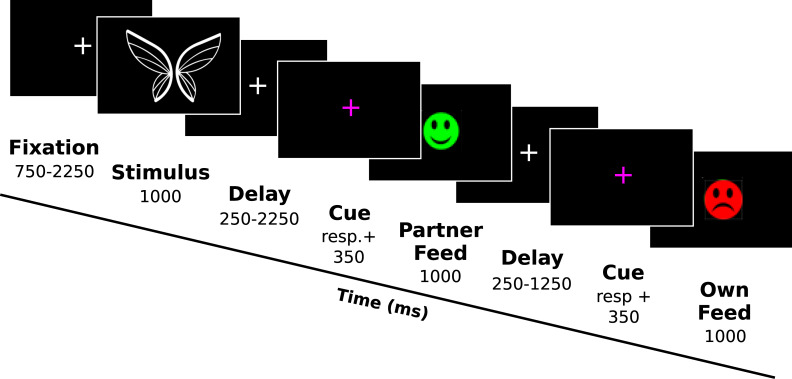
Trial sequence. The figure depicts the sequence of a single trial and the presentation time for each screen. Participants respond to two purple cue screens on every trial, once to reveal feedback for themselves and then subsequently to reveal the feedback for their partner.

Participants first saw a white fixation cross, which was presented in the centre of the display for 750–2250 ms. This was followed by a picture stimulus for 1000 ms. A second white fixation cross then appeared, which changed colour to purple after an interval that varied from 250 to 2250 ms. The purple fixation cross cue had a maximum duration of 1000 ms. Participants pressed the spacebar on a computer keyboard with the right hand in response to the cue to reveal the feedback of the other player, which was displayed for 1000 ms. Next, the white fixation cross returned for 250–1250 ms and was again replaced by a purple fixation cross. Participants pressed the spacebar for a second time in response to this cue, this time to reveal their own feedback (1000 ms). Feedback was either a happy green or sad red smiley. Participants received points for good feedback, which could be tracked on a points scale in the lower half of the screen. When the points scale was full, participants received a gold coin and the points scale was reset. If participants did not respond to either of the purple cues within their respective 1000 ms response windows, a grey circle with a line through it was displayed in place of an outcome. Neither participant earned points for ‘mistrials’ (Fig 2). Participants were informed that there was a time-window and that they should try to avoid mistrials, but the time-window itself was not specified in the instructions.

### Probe trials

To gain insight into participants’ explicit stimulus value estimates and to ensure attendance to own and observed outcomes on each trial, we asked for an estimate of the value of each stimulus from the participant, four times per block. These probe trials appeared every 15 trials. Participants were presented with an image of each stimulus in the centre of the screen and were asked to move an arrow using the up- and down-arrow keys along a vertical scale from zero (low value) to ten (high value) to indicate its estimated value. They did this twice, once to rate the value of the stimulus for themselves and once to provide an estimate of the value of the same stimulus for the other player. The ‘observer’ did not participate in probe trials.

### Test trials

To get a simple overview of participants’ performance and to motivate participants to perform the task, participants could earn extra points by correctly categorising a presented stimulus according to its value. From the point of view of the participant, this also gave the other player a logical reason to participate in the task. At the end of each block, participants were presented with one of the three stimuli from the previous block in the centre of the screen. Text above the image requested that ‘both’ players, i.e. the actor and the observer, categorise the value of this image by pressing button one (low value), two (medium value) or three (high value) on a computer keyboard. The choices were not highlighted; therefore, it was not possible for the participant to know what the other player chose. Again, participants categorised each stimulus twice, once to indicate the value of the image for themselves and again to indicate the value of the stimulus for the other player. Each correct answer earned the participant and the other player 500 bonus points. The points for the participant and the other player were displayed after each choice screen. The other player was correct, i.e. their score increased after a choice, 80% of the time. The correct response rate was chosen based on a series of pilots that preceded the experiment and was designed to be comparable to human performance in the same task. The points earned in the task could be used to enter a draw to win one of two €30 vouchers in addition to the standard pay received for participants’ time. A greater number of points meant that participants had a higher chance of their name being drawn, which added to the competitive nature of the task.

### Competitiveness questionnaire

At the end of the experiment, participants filled in the cooperativeness and competitiveness personality scale (CCPS) [23]. The questionnaire consisted of 23 items split into subdimensions that span the cognitive (8 questions), behavioural (8 questions), and affective (7 questions) domains of cooperation and competition. ‘I enjoy working with other people to achieve common success’ is an example of a question that probes positive affect related to cooperation (for further examples, see the Appendix). For each item, participants were asked to circle a number on a Likert scale (1 = completely disagree, 7 = completely agree) to indicate their agreement with the statement. From this scale we computed a single competitiveness score per participant. The reliability of the scale in the competitiveness domain has previously been found to range from .71 to .79 [24, 23]. We expected that highly competitive participants would be more prone to the effect identified by Noritake et al. [9], whereby the reward of another player influenced the perceived value of stimuli for the participant, despite outputting a constant rate of reward. Resulting competitiveness scores ranged from 1.60 to 5.80 on a 7-point Likert scale. These scores were normally distributed with a mean competitiveness score of 3.87 (*SD* = 1.01).

### EEG recording and pre-processing

Participants wore an elasticated Easycap EEG cap with an extended 10–20 system of Ag/AgCl sintered electrodes and BrainAmp MR plus amplifiers (Brain Products, GmbH, Munich, Germany). Sixty-four-channel EEG data were recorded at a rate of 500 Hz from both participants simultaneously throughout the learning task. AFz acted as the ground electrode and the reference was placed at electrode CPz. Impedances were restricted to below 5 kΩ. Additional electrodes were placed above and below the left eye and on the outer right and left canthi respectively to record vertical and horizontal eye movements. Offline analyses were conducted with custom scripts written with MATLAB 2015b (MathWorks) for EEGLAB 13.5.4b [25]. Using these scripts, the data were filtered from 0.5–40 Hz, re-referenced to common average, and epoched from −1–3.5 s after the onset of the stimulus of interest. We then pruned out epochs that contained artifacts, which were indicated by outlying activity. Fewer than 5% of trials were removed. Adaptive Mixture Independent Component Analysis (AMICA) was applied to demeaned data to identify and remove any remaining artifacts of concern. These components were visually identified by the experimenter and removed. A component was rejected if it conformed to a pattern of signal common to well-established artifacts [26], such as eye movements or muscle activity. These components were visually identified and removed.

### EEG analyses

Baseline-corrected (−200–0 ms) stimulus-locked epochs were extracted for analysis. The mean amplitude between 150–250 ms at electrode site Fz was chosen for the analysis of early stimulus processing. In previous studies that have focused on stimulus processing in learning and decision-making tasks, the P200 component has been measured within this time-window [15, 27, 28, 29, 30]. The stimulus-locked P200 has typically been measured at frontal electrode sites, of which site Fz is prevalent in the literature [27, 10, 29, 30] Therefore, this electrode and time-window were selected a priori for stimulus-locked analyses. The P200 has been linked to the dedication of attention to informative stimuli in a reinforcement learning context, hence the choice of this component as a target for analyses [10, 29, 31]. We additionally extracted demeaned outcome-locked epochs. Demeaned epochs are recommended in this case, since the time-windows between cue and feedback or response and feedback are short and can be contaminated by the anticipation of a loss or gain [31]. Two timeframes were of interest: 200–350 ms at site FCz, which corresponds to the FRN component [32, 33]; and the mean amplitude 350–500 ms at site Pz, which is a time-window and site for which the parietal P300 tends to be measured in the literature and maximal [34, 35, 36]. For our analyses, the FRN was defined as the mean difference in amplitude between gain and loss trials within the selected time-window, similar to previous studies that examine this component [37, 38, 39].

### SCR recording and preprocessing

Skin conductance response was measured with two Ag/AgCl electrodes attached to the inner side of the first and second fingers on the distal section of the left hand. Skin conductance data were sampled at a rate of 500 Hz using a BrainVision BrainAmp ExG MR16 amplifier (Brain Products, GmbH, Munich, Germany) and were divided into eight-second segments locked to the presentation of the stimulus in Brain Vision Analyzer. A 0.05 low-pass filter was also applied to remove high-frequency noise from the signal. Participant data were imported into MATLAB 2015b and averaged within a time-window from one to four seconds post-stimulus.

### Statistical analyses

Initial behavioural analyses were to confirm that participants engaged in the task by learning the stimulus values. We checked that the average performance on test trials was greater than chance (33.3%) using a one-sample *t*-test on the percentage of correct categorisations. We also ran a paired *t*-test to determine if the proportion of correct categorisations differed significantly when made for the participant or the other player. This would give an early indication as to whether participants had attended to the outcomes received by the other player. We excluded individual participants that did not perform above chance level from all subsequent analyses. Behavioural and physiological analyses that followed were conducted via a series of repeated measures ANOVAs on their respective dependent variables. For all aforementioned analyses, an alpha of .05 was the criterion for significance; and in the event of post hoc *t*-tests, a Bonferroni correction was applied, unless otherwise stated. Results of additional analyses that include age and gender as covariates are available with the data that accompanies the paper.

## 3. Behavioural results

### Test trials

To determine whether participants engaged in the task by learning the stimulus values and to rule out participants that were unable to perform the task, we examined the percentage of correct categorisations in the test trial phase. Participants correctly categorised stimuli as high-, medium- or low- reward on the majority of test trials (*M* = 67.74%, *SE* = 2.96). This performance was greater than chance-level performance of 33.3% (*t*(30) = 11.75, *p* < 10^−13^, *d* = 4.40, one-sample *t*-test). There was no significant difference in the percentage of correct categorisations when splitting categorisations by the target of the estimates (*t*(30) = −1.17, *p* = .250, *d* = −0.23, within-subjects *t*-test), i.e. estimates made for themselves (*M* = 65.73, *SE* = 3.17) or for the other player (*M* = 69.76, *SE* = 3.66). This suggested that participants attended to both their own and the other player’s outcomes in the task and that they could effectively learn stimulus values from both sources of information. We then looked at individual task performance. Two players were unable to successfully complete the test trials, as shown by a mean performance below chance on this task (both 31.25%). These two players and one participant that failed to follow the task instructions were excluded from all further analyses.

### Probe trials

The categorisation task probed the value of one randomly selected stimulus per block, but stimulus value estimates were also made by the participants for each image and player separately at four timepoints per block. This provides a clearer overview of participants’ ability to learn the stimulus values and their perception of the value for each individual stimulus that appeared per block. To determine if participants could accurately estimate the value of the stimuli in the task for themselves and for the other player we averaged stimulus value estimates across timepoints and conducted a 3-way ANOVA with the factors *Block Type* (variable, constant), *Estimate Target* (self, other) and *Stimulus Type* (low, neutral, high). There was a main effect of *Stimulus Type* on participants’ estimates (*F*(2, 54) = 71.22, *p* < 10^−16^, *η*_*p*_^2^ = .725) and an interaction between the *Stimulus Type* and *Block Type* (*F*(2,54) = 80.65, *p* < 10^−17^, *η*_*p*_^2^ = .749). We break down the two-way interaction between *Stimulus Type* and *Block Type*.

In blocks in which the stimulus value varied, there was a positive relationship between the stimulus type (Reward output: Low = 1, Neutral = 2, High = 3) and value estimates made by participants for the player for whom those stimulus values varied (*F*(1, 27) = 132.83, *p* < 10^−12^, *η*_*p*_^2^ = .831). The High-value stimulus was rated as significantly more rewarding than the Neutral- (*t*(1, 27) = 9.03, *p* < 10^−9^, *d* = 1.34) and Low-value stimuli (*t*(1, 27) = 11.53, *p <* 10^−11^, *d* = 2.50). Likewise, the Neutral-value stimuli were correctly rated as more rewarding(*t*(1, 27) = 9.88, *p* < 10^−10^, *d* = 1.41) than the Low-value stimuli. Blocks in which every stimulus had a constant 20% reward output, unsurprisingly, did not show the same level of differentiation between the three stimuli; nonetheless, there was a significant effect of the *Stimulus Type* (*F*(1, 27) = 4.47, *p* = .044, *η*_*p*_^2^ = .142) in this condition. Decomposition of the result showed that the Neutral-value stimulus was rated as significantly more valuable than the High-value stimulus in these blocks (*t*(1,27) = 4.44, *p* = .0004, *d* = 0.52). The remaining pairwise comparisons were not significant (all *p* ≥ .132). This suggests that participants were able to learn unique stimulus values for the same stimulus, on the basis of outcomes received by themselves and the other player. The stimulus effect did not depend on whom the estimate was made for (*Stimulus Type* ⨯ *Estimate Target* interaction: *F*(2, 54) = 1.31, *p* = .278, *η*_*p*_^2^ = .046; *Block Type* ⨯ *Estimate Target* ⨯ *Stimulus Type* interaction: *F*(2, 54) = 1.21, *p* = .307, *η*_*p*_^2^ = .043). Again, this indicates that participants attended to and used the feedback that was presented to inform their stimulus value estimates regardless of the actual recipient of the outcome.

### Reaction time to reveal feedback

We expected the RT to reveal feedback to reflect participants’ motivation to obtain the outcome that was to be revealed and for RTs to therefore indirectly indicate the expected value of the stimuli held by participants. To determine if such a relationship existed, we ran a 2×2×3 repeated measures ANOVA with the factors *Feed Recipient* (self, other), *Block Type* (SV, OV) and *Stimulus Type* (High, Neutral, Low) on median RTs. The results showed that, overall, responses to reveal feedback were slower when participants were revealing feedback for themselves (*M* = 421.73, *SE* = 11.91) than the other player (*M* = 390.09, *SE* = 9.47, *F*(1, 27) = 18.95, *p* < 10^−4^, *η*_*p*_^2^ = .412). This might reflect interference from processing the preceding feedback on RT, since the participant revealed their own feedback after the feedback of the other player had been revealed or a decrease in motivation to reveal own feedback given that the other player’s feedback can predict the player’s own upcoming feedback. The remaining main effects did not reach significance (all *p* ≥ .112). There were, however, significant two-way interactions between the factors *Stimulus Type* and *Block Type* (*F*(2, 54) = 5.21, *p* = .009, *η*_*p*_^2^ = .162) and *Stimulus Type* and *Feed Recipient* (*F*(2, 54) = 3.50, *p* = .037, *η*_*p*_^2^ = .115) and a three-way interaction that also reached significance (*F*(2, 54) = 6.75, *p* = .002, *η*_*p*_^2^ = .200). We focus on the breakdown of the three-way interaction.

We expected participants to take longer to reveal outcomes to the partner player as the chance of a reward for the partner increased. Instead, contrasts examining the *Block Type* and *Stimulus Type* interaction effect at each level of the *Feed Recipient* factor indicated a significant interaction for trials in which participants revealed their own outcome (*F*(1, 27) = 9.62, *p* = .004, *η*_*p*_^2^ = .263) but no interaction in which the other player’s outcome was revealed (*F*(1, 27) = 1.35, *p* = .255, *η*_*p*_^2^ = .048). A simple main effects analysis determined that the interaction was driven by a negative linear relationship between stimulus type and RT when participants revealed their own feedback in blocks in which the stimulus value varied for the other player (*F*(1, 27) = 12.04, *p* = .002, *η*_*p*_^2^ = .308). There was no stimulus value effect on RT when participants revealed their own outcome in SV blocks (*F*(1, 27) = 0.42, *p* = .524, *η*_*p*_^2^ = 0.15). Bonferroni-corrected post hoc analyses showed that participants were quicker to reveal their own feedback on trials in which images with a low chance of reward for the other participant were presented (*M* = 410.34, *SE* = 11.64), relative to trials in which a high-reward image was presented (*M* = 433.14, *SE* = 13.88, *t*(27) = 3.47, *p* = .005, *d* = 0.34). Faster reaction times to reveal own feedback when there is a higher chance of leading to a bad outcome for the other player seem counterintuitive, but in the event that a negative feedback is revealed to the computer player, the probability that a good outcome will be presented to the participant on that specific trial increases. This suggests that participants’ RTs did correspond with the expected value of the chosen stimulus when their certainty about the forthcoming outcome was high.

## 4. Physiological results

### Stimulus processing

To examine physiological markers of stimulus processing following the onset of images with different values for the participant and the other player, we input stimulus-locked ERP components and SCR signal to a GLM with the factors *Stimulus Value* (High, Medium, Low) and *Block Type* (SV, OV). If participants integrate own- and other-stimulus values similarly to the primates in Noritake et al. [9], we might expect a difference in the amplitude of components related to attentional orienting and higher-level processing for the different stimulus value types in the variable- and constant-stimulus-value conditions.

### N100 (90–150 ms, Fz)

**Fig 3:**
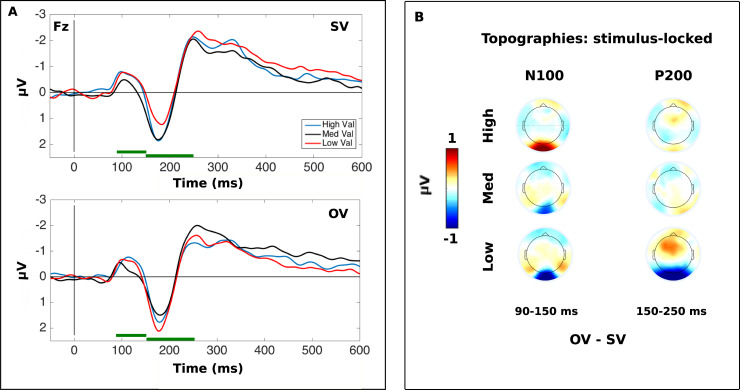
Stimulus-locked ERPs at Fz. (A) The time-course of the neural response at Fz to high, neutral and low value stimuli in SV and OV stimulus value blocks. The N100 and P200 component amplitudes are presented in the stimulus-locked analysis section. The two green bars along the x-axis highlight the N100 and P200 time-windows. (B) Scalp topographies showing the difference in amplitude between the SV and OV blocks averaged over the N100 (left) and P200 (right) time-windows.

In the N100 time-window, 90–150 ms after stimulus onset, there was a main effect of *Stimulus Type* at frontal electrodes (Fz, *F*(2, 54) = 6.055, *p* = .004, *η*_*p*_^2^ = .183). Pairwise comparisons indicated that High-reward stimuli (*M* = −0.56, *SE* = 0.23) were associated with a more negative N100 than Neutrally-rewarding (*M* = −0.20, *SE* = 0.25) stimuli (High vs. Neutral: *t*(27) = 3.39, *p* = .006, *d* = 0.28). Low-reward stimuli (*M* = −0.49, *SE* = 0.24) also provoked a more negative N100 than Neutral stimuli, but the effect did not reach significance (Low v. Neutral: *t*(27) = 2.41, *p* = .068, *d* = 0.23). The main effect of *Block Type* (*F*(1, 27) = .108, *p* = .745, *η*_*p*_^2^ = .004) and interaction between *Block Type* and *Stimulus Type*factors were not significant (*F*(2, 54) = .574, *p* = .554, *η*_*p*_^2^ = .021) (Fig 3). When competitiveness was included into the same model, there were no moderating effects.

### P200 (150–250 ms time-window, Fz)

Within the timeframe of the P200 (150–250 ms) component we found a main effect of *Block Type* (*F*(1, 27) = 16.78, *p* < 10^−4^, *η*_*p*_^2^ = .383) that indicated generally larger amplitudes in response to the stimuli when the other player’s stimulus values varied (*M* = 0.33, *SE* = 0.24) than when participants’ own stimulus values varied (*M* = 0.06, *SE* = 0.23). There was no main effect of *Stimulus Type* (*F*(2, 54) = 0.24, *p* = .976, *η*_*p*_^2^ = .001) but a significant interaction between *Block Type* and *Stimulus Type* (*F*(2, 54) = 8.40, *p* = .001, *η*_*p*_^2^ = .237). Bonferroni-corrected pairwise comparisons showed that P200 amplitudes in response to Low- (*t*(27) = −4.59, *p* < 10^−6^, *d* = 0.50) and High-value stimuli (*t*(27) = −2.25, *p* = .033, d = 0.34) differed significantly according to the *Block Type*, whereas the Neutral stimulus amplitudes did not show such an effect (*t*(27) = 0.81, *p* = .425, *d* = 0.07). Specifically, the P200 in response to Low-value stimuli was significantly greater in constant stimulus value blocks (*M* = 0.51, *SE* = 0.26) than in variable-stimulus-value blocks (*M* = −0.14, *SE* = 0.24). The High-value stimuli also provoked a significantly larger P200 in OV blocks (*M* = 0.07, *SE* = 0.25) than the SV blocks (*M* = 0.33, *SE* = 0.25).

**Fig 4:**
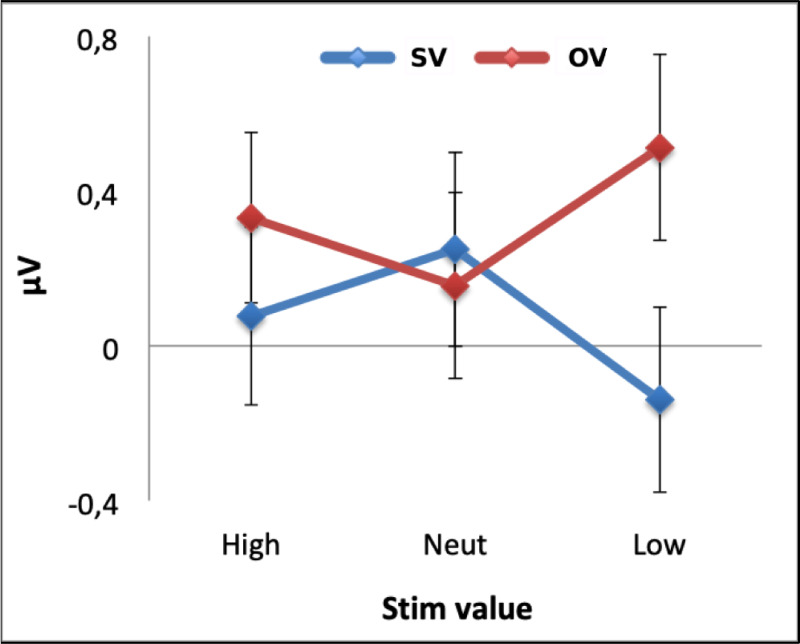
Mean P200 response at Fz. The figure depicts P200 component mean amplitudes in response to High-, Neutral- and Low-value stimuli plotted separately for the SV and OV blocks. The bars represent standard error.

Within the self-variable condition, the P200 in response to Low-value stimuli (*M* = −0.14, *SE* = 0.24) was significantly smaller than to the Neutral-value stimuli (*M* = 0.25, *SE* = 0.23, *t*(27) = 2.96, *p* = .019, *d* = 0.32). In contrast, in the constant value condition, amplitudes to Low-value stimuli (*M* = 0.51, *SE* = 0.26) were significantly greater than to the neutral value stimuli (*M* = 0.16, *SE* = 0.24, *t*(27) = −2.77, *p* = .030, *d* = 0.28). None of the remaining effects were significant (all *p* > .299 corrected) (Fig 4). Again, the addition of participants’ competitiveness scores to the same model did not indicate any moderating effects. These results indicate that the stimulus-locked P200 component amplitude is modulated by stimulus value, but are not able to exclude a possible impact of the N100 on the P200 component amplitude.

### Skin conductance response (1–4 s after stimulus)

SCR as a measure of arousal can reflect the subjective value or risk associated with a stimulus: therefore, we examined the SCR to each stimulus type for blocks in which the stimulus varied or remained constant for the participants. Again we applied a model with the factors *Block Type* (SV, OV), *Stimulus Type* (high, medium, low) and their interaction. SCR values were taken as the average response 1–4 s after stimulus presentation. There was a main effect of *Block Type* (*F*(1, 27) = 4.38, *p* = .046, *η*_*p*_^2^ = .140), in which the SCR was greater overall in SV (*M* = −1.74, *SE* = 0.38) than OV stimulus value blocks (*M* = −2.36, *SE* = 0.43), a main effect of *Stimulus Type* (*F*(2, 54) = 4.25, *p* = .019, *η*_*p*_^2^ *=* .136) and an interaction between *Stimulus Type* and *Block Type (F*(2, 54) = 8.72, *p* = .001, *η*_*p*_^2^ = .244). When stimulus values varied for the participant, the Low-value stimulus provoked a significantly greater SCR than Neutral (*t*(27) = 4.13, *p* = .001, *d* = 1.00) and High-value stimuli (*t*(27) = 2.99, *p* = .013, *d* = 0.69). The High and Neutral stimuli did not differ significantly (*t*(27) = 1.58, *p* = .378, *d* = 0.21). Within the other-variable stimulus value condition, there were no significant differences between the stimulus types (all *p* = 1.00, corrected).

**Fig 5:**
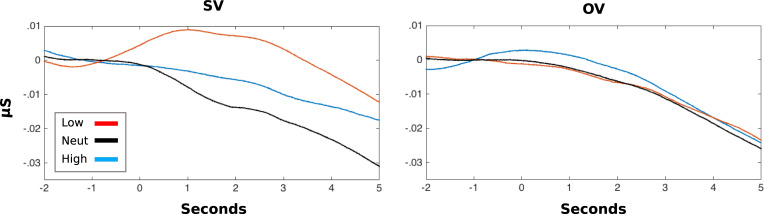
Stimulus-locked skin conductance response. The figure depicts the skin conductance response to high, medium and low values stimuli presented split by blocks type, SV or OV.

Looking at SCR in the SV and OV blocks, there was a significant difference in the amplitude of the SCR to low reward stimuli (*t*(27) = 3.33, *p* = .003, *d* = 0.92). In the SV condition, the stimulus with a low chance of reward for the participant (*M* = −0.45, *SE* = 0.34) provoked a significantly greater SCR than in the OV condition (*M* = −2.54, *SE* = 0.52). There was no significant difference in the amplitude of SCRs to Neutral- and High-value stimuli in the two block types (Fig 5). Inclusion of competitiveness into the model did not result in any significant moderating effects. SCRs evoked in this task show a pattern similar to participants’ subjective stimulus value ratings from the probe trials. They correspond well with ratings of value for themselves and do not appear to be influenced by the value of the stimuli for the other player.

### Outcome processing

#### FRN (mean difference waves 200–350 ms)

**Fig 6:**
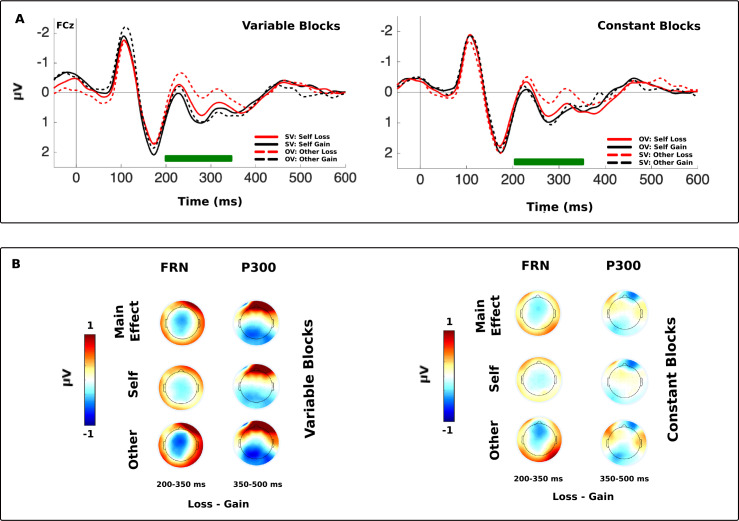
Outcome-locked ERP at FCz and outcome-locked scalp topographies. (A) ERPs plotted according to whether stimulus values varied or output a constant rate of reward and according to the recipient of the outcome. ‘Variable Blocks’ refers to blocks in which stimulus values varied and the plotted ERP is in response to the player for which the stimulus value is varying. ‘Constant Blocks’ refers to blocks in which stimuli output a constant rate of reward and the ERP is in response to the player for which the stimulus values are outputting this constant reward rate. A green bar along the x-axis highlights the FRN time-window. (B) Scalp topographies averaged across the FRN and P300 time-windows. Depicted in the first row is the overall mean difference between loss and gain within each block. In the second and third rows the mean loss-gain response to self- and other-outcomes are plotted.

To determine if differences in outcome processing existed for feedback that was personally received or given to the other player, we examined the amplitude of the FRN data for these conditions in the variable and constant reward blocks. Self-FRN and other-FRN amplitudes were computed as the subtraction of gain from loss trials averaged within a 200–350 ms time-window for each condition at site FCz. An ANOVA with the factors *Feedback Type* (Own, Other), *Block Type* (Variable, Constant) and their interaction was applied to this data (Fig 6). The data for all outcome-locked analyses were deameaned but a −50–0 ms pre-stimulus baseline yielded qualitatively comparable results.

When participants observed the other player receive feedback (*M* = −0.61, *SE* = 0.09) the other-FRN in response to the other player’s feedback appeared greater than the self-FRN that was received when participants viewed their own feedback (*M* = −0.49, SE = 0.09); this effect was significant (*F*(1, 27) = 11.21, *p* = .002, *η*_*p*_^2^ = .293). There was no significant main effect of *Block Type* on FRN amplitude (*F*(1, 27) = 2.12, *p* = .157, *η*_*p*_^2^ = .073).

**Fig 7:**
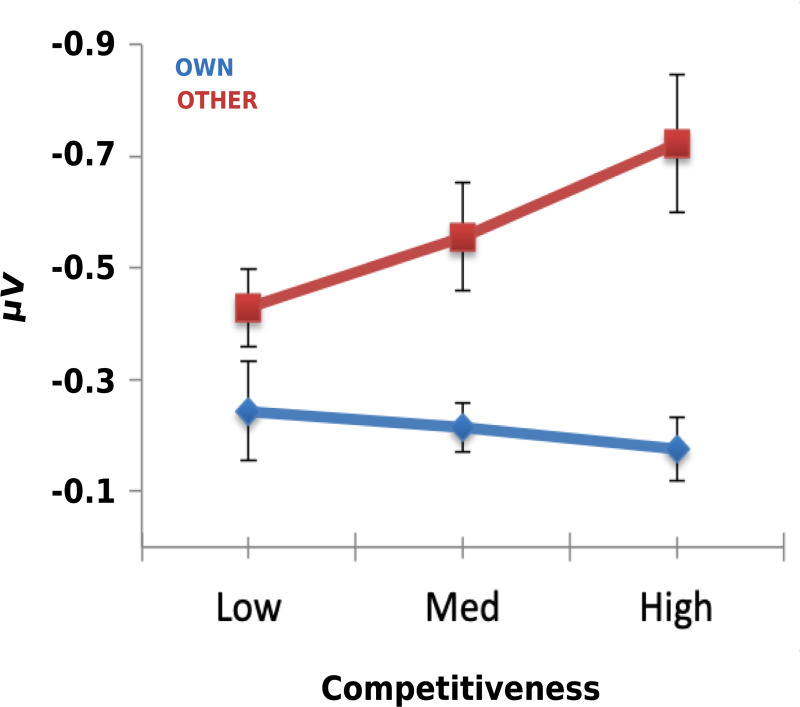
Outcome-locked FRN modulation by self-reported competitiveness. Each plot depicts the FRN difference wave amplitude to own (blue) and partner (red) outcome across block types. The data are then split by competitiveness score. Low, medium and high competitiveness refers to scores that fell within the lowest, medium and highest quartile of self-reported competitiveness scores respectively. The bars represent standard error.

Although the difference between the amplitude of the other-FRN and self-FRN appeared greater in blocks in which stimulus values varied for the recipient of the feedback, there was no significant interaction between the *Feedback Type* and *Block Type* factors’ amplitude (*F*(1, 27) = 1.51, *p* = .230, *η*_*p*_^2^ = .053). The amplitude of the FRN is typically reduced for observed feedback, but in this experiment the feedback of the other player was shown first and partially predicted the feedback of the participant, which was to follow. This may have impacted on the amplitude of the other-FRN, by changing how predictable a gain or loss could be on a given trial. The inclusion of participants’ competitiveness score into the same model indicated that the amplitude of the other-FRN and self-FRN were influenced by participants’ competitiveness. There was a significant two-way interaction between the factors *Feed Type* and *Competitiveness* (*F*(1, 26) = 6.33, *p* = .018, *η*_*p*_^2^ = .196). FRN difference wave amplitude increased in line with participants’ trait competitiveness (Fig 7). There was no further influence of competitiveness on participants’ FRN amplitudes.

#### P300 (350–500 ms time-window, FCz and Pz)

Next we examined the effects of the same regressors on the amplitude of the P3a and P3b components at site FCz and Pz respectively. An additional regressor coded for *Feedback Valence* (Gain, Loss). On average, P3a amplitude was greater for gains (*M* = 0.08, *SE* = 0.19) than losses (*M* = 0.04, *SE* = 0.19), but the effect was not significant (*Feed Valence*: *F*(1, 27) = 0.30, *p* = .590, *η*_*p*_^2^ = .011). Mean P3a amplitude differed according to *Block Type* in that there was a larger mean P3a amplitude when stimulus values varied (*M* = 0.09, *SE* = 0.18) compared to when a constant rate of reward was output (*M* = 0.04, *SE* = 0.19), but again this did not reach significance (*F*(1, 27) = 3.07, *p* = .091, *η*_*p*_^2^ = .102). There were no other effects of note within this time-window at FCz (all *P* ≥ .175); nor did competitiveness scores interact with any of the effects.

At Pz, the parietal P3b in the same timeframe was also greater for gains (*M* = 0.64, *SE* = 0.29) than losses (*M* = 0.28, *SE* = 0.28), but this time the effect was significant (*F*(1, 27) = 14.46, *p* = .001, *η*_*p*_^2^ = .349). Typically the P3b is greater for losses than gains; therefore, this result is unexpected. There was also a significant main effect of *Feed Type*, that showed that the P3b amplitude in response to the other player’s feedback (*M* = 0.50, *SE* = 0.28) was greater than to participants’ own feedback (*M* = 0.41, SE = 0.28, *F*(1, 27) = 4.29, *p* = .048, *η*_*p*_^*2*^ = .137). There was no main effect of *Block Type* (*F*(1, 27) = 2.02, *p* = .167, *η*_*p*_^2^ = .069), but there was a significant two-way interaction between the *Block Type* and *Feed Type* factors (*F*(1, 27) = 5.32, *p* = .029, *η*_*p*_^2^ = .164). The P3b response to the other player’s feedback (*M* = 0.55, *SE* = 0.28) was greater than the response to participants’ own feedback (*M* = 0.32, *SE* = 0.31) when stimulus values output a constant rate of reward (*t*(27) = 2.71, *p* = .011, *d* = 0.18), but did not differ significantly when stimulus values varied (Own: *M* = 0.51, *SE* = 0.25; Other: *M* = 0.46, *SE* = 0.29; *t*(27) = 0.82, *p* = .418, *d* = 0.04). There was also a significant interaction between the *Feed Type* and *Feed Valence* regressors (*F*(1, 27) = 5.82, *p* = .023, *η*_*p*_^2^ = .177). P3b amplitude in response to gains made by the other player were significantly greater than to participants’ own gains (Own: *M* = 0.52, *SE* = 0.28; Other: *M* = 0.76, *SE* = 0.31; *t*(27) = 2.58, *p* = .016, *d* = 0.19), whereas the P3b amplitudes were similar for own and other losses (Own: *M* = 0.31, *SE* = 0.29; Other: *M* = 0.25, *SE* = 0.28; *t*(27) = 1.15, *p* = .259, *d* = 0.05). An interaction between *Feed Valence* and *Block Type* did not quite reach significance (*F*(1, 27) = 3.81, *p* = .061, *η*_*p*_^2^ = .124), but generally it appeared that there was a greater difference between P3b amplitudes to gains and losses when stimulus values varied (Gain: *M* = 0.77, *SE* = 0.24; Loss: *M* = 0.20, *SE* = 0.32), compared to when stimulus values output a constant reward rate (Gain: *M* = 0.51, *SE* = 0.34; Loss: *M* = 0.35, *SE* = 0.25). Although unusual, a greater P3b to gains compared to losses would be consistent with the idea that the P3b reflects surprise, since gains for the participant within OV blocks and the other player in SV blocks were rare events.

## 5. Discussion

### Summary

Multiple investigations show that the interpretation of reward is dependent on the social context in which it is received [3, 1, 6]. In the present study we used ERPs and SCR to examine the timecourse of the representation of outcomes and stimulus values that were retrieved while learning in a competitive social context. We additionally measured trait competitiveness to determine if individual differences in competitiveness altered behavioural and physiological responses to own and other outcomes and stimuli of different incentive value.

### Stimulus value estimates: RT & behaviour

We expected that in a competitive social context, participants would be slower to reveal their partner’s feedback when a reward for that partner was likely, i.e. RT could reflect reluctance for the other player to receive a reward due to the corresponding reduction in expectation of reward for the self. This would be similar to the licking behaviour seen by Noritake et al. [9] in which high value stimuli for a partner monkey in OV blocks were associated with a reduction in licking frequency on a water tube. However, contrary to work by Noritake et al. [9], RTs to reveal the other player’s feedback in OV blocks did not depend on the value of the stimulus for the partner player. Two potential explanations for this finding are 1) that participants did not perceive the task to be sufficiently competitive to provoke a preference towards stimuli that output a low rate of reward for the other player and 2) that probe trials, which interrupted the learning trials, may have made it easier for participants to maintain a clear perception of the actual value of the stimuli for the self and other in mind, thus preventing the development of a bias.

In contrast, RTs to reveal own feedback in OV blocks did depend on stimulus value for the other player. High value stimuli in OV blocks were associated with slower RTs to reveal own feedback than low value stimuli. This suggested that, regardless of whether or not participants’ perception of value was biased by the competitive structure of the task, participants did recognise that a positive outcome for the other player and themselves never appeared consecutively and that their RTs corresponded with their subjective expectation of reward on a trial-by-trial basis. This is in line with evidence that there is an inverse relationship between subjective expectation of reward and RT [12, 19].

### The stimulus-locked N100 is biased by learned stimulus value/salience

With respect to the physiological findings, in the stimulus-locked analyses, we see an initial modulation of the N100 component by stimulus value around 90–150 ms post-stimulus at frontal electrode sites. High value stimuli provoked a greater response than the neutral stimulus across SV and OV blocks, while low-value stimuli provoked a weaker increase in N100 amplitude relative to the neutral stimulus that did not reach significance. Early visual components occurring less than 200 ms post-stimulus are typically associated with rapid, involuntary stages of exogenous attentional processing that occur to stimuli that are naturally relevant for the viewer, such as food [40] but also to focused attention to valuable stimuli that have been learned over shorter intervals [41, 42, 43, 44]. This could be the case for the high-value stimuli in our task. We found no evidence that stimulus processing differed according to block type at this early stage of processing, but this is entirely expected. Higher order goals and strategies associated with behaving in a competitive environment would be unlikely to be represented in initial stages of stimulus evaluation.

### Stimulus-locked P200 component amplitude is modulated by stimulus value

We confirmed that in blocks in which stimulus value varied for the participant, P200 amplitude 150–200 ms post-stimulus varied according to stimulus value at frontocentral electrodes. High- and medium-value stimuli provoked a significantly greater P200 response than low-value stimuli. In accordance with Luque et al. [10], we might consider this signal to reflect the response-predictive value of the stimulus, i.e. the extent to which the stimulus is associated with a response and therefore commands attention due to its importance in prompting an upcoming action. Approximately 300–500 ms post-stimulus presentation the P3a at Fz was also modulated by stimulus value, but the effects were less clear. The neutral- and high-value stimuli provoked a more positive response than the low-value stimuli but only the low- and neutral-value stimuli signals differed significantly.

In the study conducted by Luque et al. [10], in which they disentangled response and reward prediction, the authors concluded that while P200 amplitude corresponded with response-predictive value, it was the P3a that was associated with reward-predictive value. Since participants did not make a choice in this study but rather were required on every trial to respond by pressing to reveal feedback, the design does not distinguish between response and reward prediction effects. In our task the high-value stimulus could provoke a larger P200 than the low-value stimulus, either because it is more strongly coupled with the button press action or because it is more associated with a positive outcome. However, there are other studies that show that P200 amplitude reflects attention dedicated to the stimulus on account of its reward-predicting properties [16, 18]. Therefore, the P200 effects seen here could equally be consistent with this literature.

### Stimulus-locked P200 and P3a stimulus value effects are modulated by social context

What was of particular interest was that social comparison impacted stimulus processing in the P200 and P3a timeframes. In OV blocks, in which stimuli varied in value for the other player but output a constant rate of reward for the participant, the mean amplitude of the P200 was greatest in response to stimuli with a low value for the other player but greater subjective value for the participant. The change in P200 amplitude to low-value stimuli between SV and OV blocks was significant, but the P200 to high-value stimuli was also greater in OV compared to SV blocks, and the low- and high-value stimulus P200 did not differ significantly following correction for multiple comparisons. Contrary to our expectation, there was also no effect of trait competitiveness on the extent that high- and low-value stimuli modulated the P200 in the OV and SV blocks. The P3a amplitudes to stimuli of differing values followed the same pattern as the P200 effects. In contrast to Noritake et al. [9], for whom clear social comparison-like behaviour were found in the neurophysiological and behavioural data of non-human primates, we can only confirm that the block type had an effect on the processing of the high- and low-value stimuli. Nonetheless, the pattern of effects we see, in which stimulus evaluation in OV blocks resembles an inversion of the SV block effects, is in line with what we would expect if participants’ stimulus evaluation is impacted by social comparison.

### The outcome of the other player is more important to competitive participants

Although the competitive social context did not conclusively impact stimulus evaluation, outcome-locked analysis of the FRN component 250–300 ms post-outcome was modulated by trait competitiveness. Specifically, other-FRN amplitudes increased in line with participants’ trait competitiveness scores. The FRN is sensitive to motivational significance and attention; therefore, the finding may reflect increased cognitive resources dedicated to processing outcomes in the task by participants as they become more invested in their own performance relative to the other player. These results are consistent with a recent study in which participants observed outcomes for an opponent and a partner in a competitive gambling task [6]. The authors found a larger FRN in response to the opponent’s loss compared to the partner’s loss, and considered whether participants were engaging in perspective-taking to ‘know their enemy’ and thus confer a competitive advantage for themselves during the experiment. In that study, the other-FRN correlated with self-reported measure of self-other overlap, thereby providing additional evidence for their conclusion.

### Motivational differences between species likely impacted the competitiveness manipulation

It is somewhat unsurprising that our data are less conclusive than the results produced from the non-human primates in the study by Noritake et al. [9]. While we attempted to motivate human participants to perform the task using a points-based system leading to a real monetary reward, this is unlikely to compare to the motivation to receive water in a dehydrated state, which is the context in which non-human primates, including those performing the task in the study by Noritake et al. [9], typically learn in experimental settings [45]. The task itself was also designed to be as similar as possible to the original study, which meant that it was somewhat boring for human participants, despite several adjustments to the difficulty of the task during piloting. In contrast, for the non-human primate subjects, performance of the task is a comparably socially and cognitively enriching activity. We expect that the primary difficulty in this instance was motivational, since although participants did not appear to be strongly driven by the competitive aspect of the task, there was a high rate of adequate learning, which suggests that they engaged with aims of the experiment.

## 6. Conclusion

We find evidence for a neurophysiological effect of a competitive social context at the time of stimulus processing approximately 200 ms post-stimulus in a social associative learning task. Stimuli were evaluated differently by participants in blocks in which they received few rewards, relative to a partner for whom reward rates varied according to the presented stimulus. Participants’ trait competitiveness impacted outcome evaluation, such that the FRN to the partner’s outcome was greater in competitive participants, thereby suggesting that the outcome was of greater relevance to these individuals. Follow-up work should include recruiting of more participants to confirm results and bolstering of the competitive features of the task further to increase the perception of resource scarcity in human participants. This may lead to results more consistent with work in non-human primates. Modelling the mechanisms that lead to participants’ stimulus value estimates in the self-reported probe trials may also offer insight into the development of biases that may be provoked by decisions made in resource scarce environments.
